# AMP-activated protein kinase modulates tau phosphorylation and tau pathology *in vivo*

**DOI:** 10.1038/srep26758

**Published:** 2016-05-27

**Authors:** Manon Domise, Sébastien Didier, Claudia Marinangeli, Haitian Zhao, Pallavi Chandakkar, Luc Buée, Benoit Viollet, Peter Davies, Philippe Marambaud, Valérie Vingtdeux

**Affiliations:** 1 Univ. Lille, Inserm, CHU Lille, UMR-S 1172 – JPArc – Centre de Recherche Jean-Pierre AUBERT, F-59000 Lille, France; 2 Litwin-Zucker Research Center for the Study of Alzheimer’s disease, The Feinstein Institute for Medical Research, Manhasset, New York 11030, USA; 3 Institut Cochin, Inserm U1016, Paris 75014, France; 4 CNRS, UMR 8104, Paris 75014, France; 5 Université Paris Descartes, Sorbonne Paris Cité, Paris 75014, France

## Abstract

Neurofibrillary tangles (NFTs) are the pathological hallmark of neurodegenerative diseases commonly known as tauopathies. NFTs result from the intracellular aggregation of abnormally and hyperphosphorylated tau proteins. Tau functions, which include the regulation of microtubules dynamics, are dependent on its phosphorylation status. As a consequence, any changes in tau phosphorylation can have major impacts on synaptic plasticity and memory. Recently, it has been demonstrated that AMP-activated protein kinase (AMPK) was deregulated in the brain of Alzheimer’s disease (AD) patients where it co-localized with phosphorylated tau in pre-tangle and tangle-bearing neurons. Besides, it was found that AMPK was a tau kinase *in vitro*. Here, we find that endogenous AMPK activation in mouse primary neurons induced an increase of tau phosphorylation at multiple sites, whereas AMPK inhibition led to a rapid decrease of tau phosphorylation. We further show that AMPK mice deficient for one of the catalytic alpha subunits displayed reduced endogenous tau phosphorylation. Finally, we found that AMPK deficiency reduced tau pathology in the PS19 mouse model of tauopathy. These results show that AMPK regulates tau phosphorylation in mouse primary neurons as well as *in vivo*, and thus suggest that AMPK could be a key player in the development of AD pathology.

AMP-activated protein kinase (AMPK) is a master energy sensor whose activation following energy stress conditions helps maintain cellular energy levels. In this context, AMPK acts by phosphorylating key enzymes in metabolic pathways as well as transcriptional factors and cofactors. AMPK is a hetero-trimeric complex composed of a catalytic α subunit and two regulatory β and γ subunits. Several genes encode these subunits (α1, α2, β1, β2, γ1, γ2, γ3) giving rise to a multitude of complexes. The regulatory γ subunit possesses four CBS (cystathionine-beta-synthase) domains from which three of them are able to bind adenine nucleotides thereby allowing the kinase to sense and respond to AMP, ADP and ATP intracellular levels. In particular, AMP binding induces a conformational change in the α-subunit activation loop of the kinase that allows phosphorylation of the activating residue Thr^172^ by AMPK upstream kinases. Two main upstream kinases have been reported: LKB1 (liver kinase B1) and CaMKKβ (Ca^2+^/calmodulin dependent kinase kinase β). Although in the brain AMPK is mainly expressed in neurons^1^,^2^, its role in neuronal homeostasis and during neurodegeneration remains to be clearly established. In humans, it is becoming evident that AMPK is deregulated in major neurodegenerative diseases, including Huntington’s^3^, Parkinson’s^4^ and tauopathies^5^. It is acknowledged that the two α subunits of AMPK can have distinct roles. Indeed, it has been demonstrated that AMPKα2, which is the most highly expressed of the catalytic subunit in the brain^2^, was responsible for the detrimental effects observed following ischemic stroke in mice^6^. Whereas, AMPKα1 deregulation seems to play a key role in Huntington’s disease^3^.

Tauopathies - the best known of them being Alzheimer’s disease (AD) - are a subset of neurodegenerative diseases characterized by a common pathological process, aggregation of the microtubule-associated tau protein. Among these disorders, such aggregation leads to neurofibrillary tangles (NFTs), Pick bodies or argyrophilic grains. In all of these histopathological hallmarks, tau proteins are found abnormally and hyper-phosphorylated. Tau main functions include microtubules stabilization, neurite outgrowth, and facilitation of organelles axonal transport including mitochondria^7^,^8^,^9^. These functions are regulated by tau phosphorylation status, for instance, residues Ser^262^ and Ser^356^ phosphorylation was shown to alter the microtubule binding properties of tau^10^. In addition tau phosphorylation is associated to changes in synaptic plasticity and memory^11^,^12^,^13^. As a consequence, a disequilibrium between tau kinases and phosphatases activities is believed to play a central role in the development of AD. In this context, several kinases have been reported to phosphorylate tau including glycogen synthase kinase 3 (GSK-3α and β)^14^,^15^,^16^, cyclin dependent kinase 5 (cdk5)^16^,^17^,^18^, protein kinase A (PKA)^19^, mitogen-activated protein kinases (MAPK)^20^ and the MARK family of protein kinases^21^,^22^. We and other also demonstrated that AMPK is a tau kinase *in vitro*^5^,^23^. In addition, activated AMPK is found accumulated in pre-tangle- and tangle-bearing neurons in AD and more largely in tauopathies. Further, expression levels of AMPK have been quantitatively shown to be elevated in AD^24^. The potential importance of AMPK in AD development is also supported by recent evidence. It was found that Amyloid-β (Aβ) oligomers, which are the core components of senile plaques, the other hallmark of AD, induce a rise in intracellular Ca^2+^ concentration thus leading to CaMKKβ activation and subsequent AMPKα1 phosphorylation^23^,^25^. In these conditions, AMPK was suggested to mediate the toxic effects of Aβ exposure on dendritic spine numbers^25^ and long-term potentiation (LTP)^24^. LTP impairments were also reported following AMPK activation mediated by AICAR (5-aminoimidazole-4-carboxamide ribonucleotide), 2-deoxy-D-glucose and metformin treatments^26^. However, the direct role of AMPK on tau phosphorylation in cell models remains controversial^27^,^28^ and the effect of AMPK activation mediated by metabolic stress on tau phosphorylation remains to be established. Indeed, metabolic impairments, including glucose hypometabolism and mitochondrial defects, are another early feature of AD that could participate to AMPK activation^29^,^30^,^31^.

The goal of our study was to determine whether AMPK is involved in tau phosphorylation in differentiated primary neurons and *in vivo*. To this end, we used a pharmacological approach to mimic energetic stress conditions to activate AMPK. We then evaluated the involvement of AMPK *in vivo* in the mouse brain by assessing endogenous tau phosphorylation levels in the brain of mice conditionally knock-out (KO) for the α2 subunit of AMPK and in AMPKα2 KO mice crossed with the PS19 tau transgenic mouse model of tauopathy.

## Results

### AMPK interacts with tau in neurons

**Figure 1:**
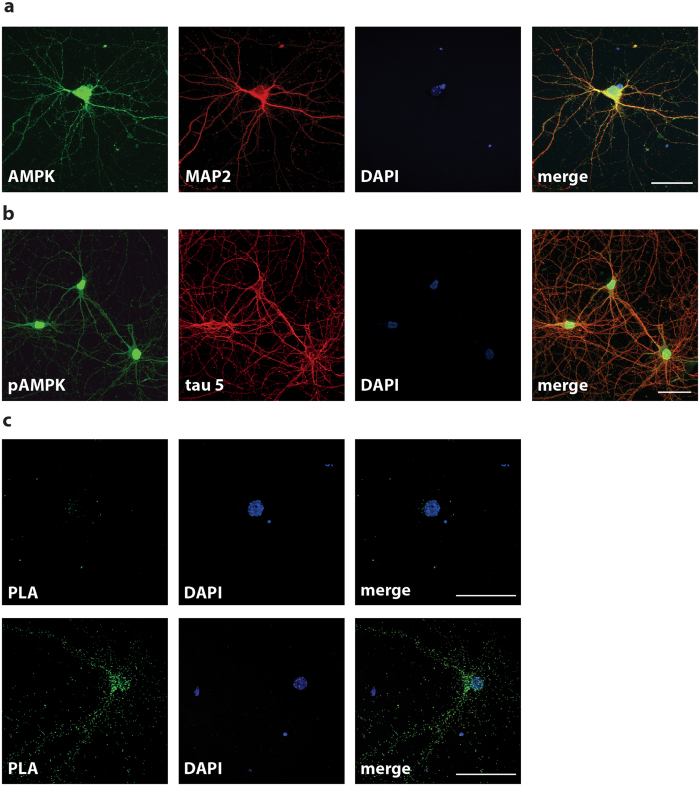
AMPK co-localizes and interacts with tau in primary neurons. (**a,b)** Immunofluorescence staining of total AMPK (AMPKα1/α2, green) and MAP2 (red) (**a**) or of activated AMPK (p-Thr^172^AMPK, green) and total tau (Tau5, red) (**b**) in primary neurons at 15 DIV. (**c**) PLA in primary neurons at 15 DIV using activated AMPK (p-Thr^172^AMPK) and total tau (Tau5) primary antibodies. PLA negative control (top panel). Green spots indicate interactions between AMPK and tau (bottom panel). Scale bars = 50 μm.

In the brain, AMPK and tau are mainly expressed in neurons therefore we used differentiated primary neurons as a model to perform our experiments. First, we determined the subcellular localization of AMPK and tau in our model. Total AMPK was localized in dendrites, cell body and nucleus (Fig. 1a), as previously reported^5^,^32^. Tau and activated AMPK (phosphorylated at Thr^172^) co-localized both in neurites and cell body (Fig. 1b). Primary neurons treated with the AMPK activator AICAR showed an increase of phosphorylated AMPK staining as compared to the control condition (Fig. S1a) while AMPK staining was completely abolished after incubation with an antibody blocking peptide, thus showing the specificity of the p-Thr^172^AMPK antibody (Fig. S1b). To further analyze the co-localization of tau with AMPK, we performed duolink proximity ligation assay (PLA) to detect *in situ* protein interactions using anti-tau5 (total tau) and anti-p-Thr^172^AMPK primary antibodies. PLA amplification occurs only if the antigens are located within 40 nm of proximity. PLA staining which is visualized as fluorescent dots was observed in cell body and neurites thereby reflecting AMPK-tau interactions (Fig. 1c). Together, these results suggest that endogenous AMPK and tau co-localize and interact in neurons supporting the notion that tau could be a neuronal target of AMPK.

### AMPK activation increases tau phosphorylation in primary neurons and affects tau binding to microtubules

We and others have previously demonstrated that AMPK could phosphorylate tau *in vitro* at several epitopes including Thr^231^, Ser^262/356^, Ser^396^, Ser^409^ and Ser^422^; whereas Ser^199^, Ser^202^, Ser^235^, Ser^400^, and Ser^404^ were not found to be direct AMPK substrates^5^,^23^. To study the involvement of AMPK on tau phosphorylation in a neuronal model, we used primary neuronal cultures at 15 DIV. At this differentiation time point, tau staining in Western-blot appears as two distinct bands corresponding to the two isoforms of tau containing 3 or 4 microtubules binding domains (3R and 4R isoforms). Neurons were challenged with drugs known to activate AMPK by mimicking an energy stress condition that are AICAR, and metformin. AICAR is a cell-permeable nucleoside that is metabolically converted by adenosine kinase to AICA ribotide (ZMP), a purine precursor that acts as a 5′-AMP analogue whereas metformin activates indirectly AMPK through LKB1 by acting as a mild inhibitor of the respiratory chain Complex I, subsequently leading to a drop of intracellular ATP levels^33^.

**Figure 2:**
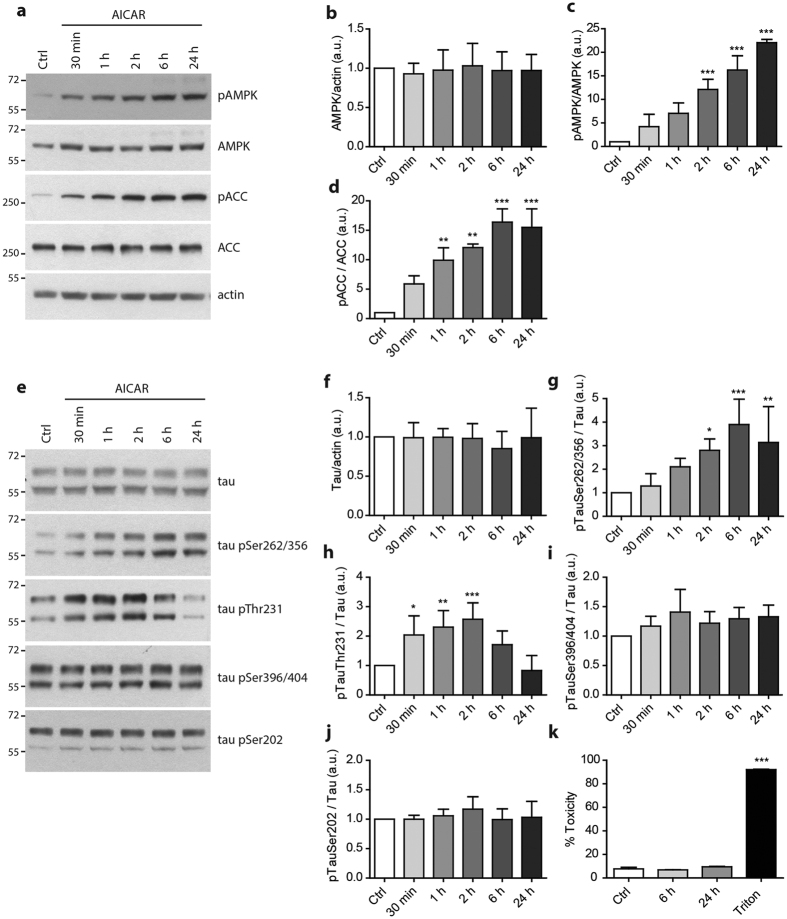
AICAR-mediated AMPK activation induces tau phosphorylation in primary neurons. Primary neurons at 15 DIV were treated for the indicated times with the AMPK activator AICAR (1 mM). AMPK and ACC expression and activation were monitored by Western-blot (WB) using antibodies against AMPK, p-Thr^172^AMPK (pAMPK), ACC, p-Ser^79^ACC (pACC) and actin (**a**). Quantifications of the ratios AMPK/actin (**b**) pAMPK/AMPK (**c**) and pACC/ACC (**d**). WB analysis (**e**) and quantification of total tau (**f**) and phosphorylated tau at epitopes Ser^262/356^ (**g**), Thr^231^ (**h**), Ser^396/404^ (**i**) and Ser^202^ (**j**) expressed in ratios. Cytotoxicity assay following 6 h and 24 h AICAR treatments in primary neurons at 15 DIV determined using the lactate dehydrogenase (LDH) test (**k**). Treatment with 0.9% Triton X-100 was used as a positive control. Results represent mean ± SD, n = 4–6. a.u., arbitrary units. *p < 0.05, **p < 0.01, ***p < 0.001 compared to Ctrl, One way ANOVA with Bonferroni’s post hoc test.

Primary neurons were treated with AICAR and metformin for up to 24 hours without inducing any toxicity (Fig. 2k and Fig. S2k). As expected, AICAR and metformin induced AMPK activation as shown by the increased phosphorylation of AMPK at Thr^172^ and of acetyl-CoA carboxylase (ACC) at Ser^79^, a direct cytosolic target of AMPK (Fig. 2a–d and Fig. S2a–d). Importantly, AMPK activation led to a significant increase of tau phosphorylation at several epitopes including Ser^262/356^ and Thr^231^, while phosphorylation at Ser^202^ was not changed (Fig. 2e–j and Fig. S2e–j). Similar results were obtained following metformin treatment (Fig. S2). Overall our results show that AMPK activation increased tau phosphorylation at epitopes relevant to what was previously reported *in vitro*.

**Figure 3:**
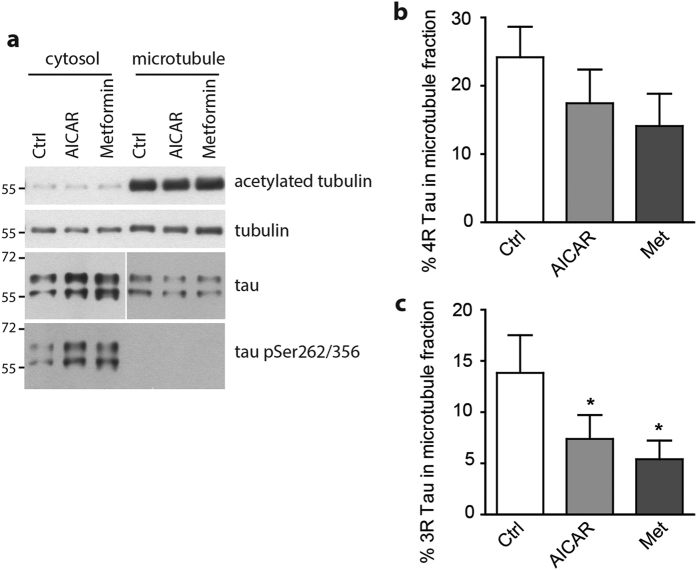
AMPK-mediated tau phosphorylation impairs tau affinity with microtubules. Primary neurons were treated with AICAR (1 mM) or metformin (2.5 mM) for 2 h prior to being subjected to cell fractionation into cytosol and microtubule fractions. WB analysis on cytosol and microtubule fractions of acetylated-tubulin, tubulin, total tau and pSer^262/356^ tau (**a**). Percentage of 4R (**b**) and 3R (**c**) tau proteins observed in the microtubule fraction upon treatment with AICAR or meformin. Results represent mean ± SD, n = 5. *p < 0.05, compared to Ctrl, One way ANOVA with Bonferroni’s post hoc test.

We next assessed the consequences of AMPK-mediated tau phosphorylation on tau ability to bind microtubules. To investigate the effect of AMPK activation on tau binding to microtubules, we used a microtubule fractionation assay^34^. Following this assay, free tau is recovered in the cytosol fraction whereas microtubule-associated tau is present in the microtubule fraction. Upon AMPK activation using AICAR or metformin, tau levels in the microtubule fraction were significantly decreased (Fig. 3) therefore suggesting that AMPK-mediated phosphorylation of tau affected its interaction with microtubules.

### AMPK inhibition decreases tau phosphorylation in neurons

**Figure 4:**
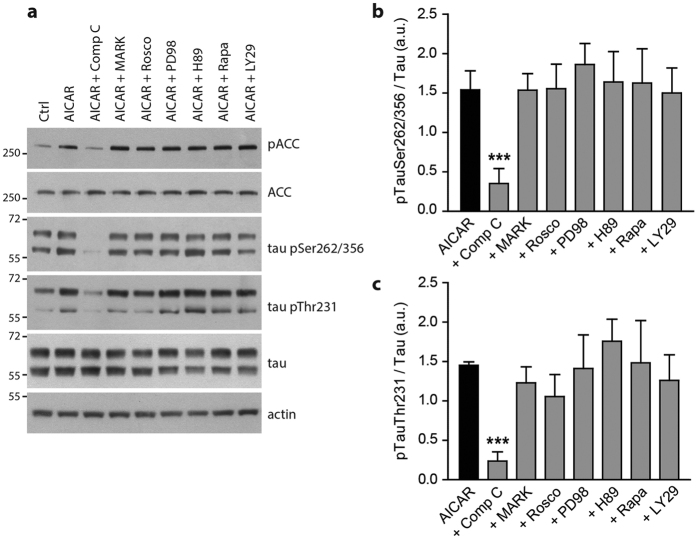
Effect of main tau kinases inhibition on AICAR-mediated tau phosphorylation. Primary neurons at 15 DIV were pre-treated (30 min) with various kinases inhibitors including, Compound C (Comp C, 10 μM), MARK/Par1 (20 μM), roscovitine (Rosco, 10 μM), PD98059 (PD98, 10 μM), H-89 (20 μM), Rapamycin (Rapa, 10 nM) and LY294002 (LY29, 20 μM) prior to AICAR treatment (1 mM, 2 h). Cell lysates were analyzed by WB for ACC, p-Ser^79^ACC (pACC), phosphorylated tau at epitopes Ser^262/356^ and Thr^231^, total tau and actin (**a**). Quantification of phosphorylated tau at epitopes Ser^262/356^ (**b**), and Thr^231^ (**c**) expressed in ratio of Ctrl. Results represent mean ± SD, n = 4. a.u, arbitrary units. ***p < 0.001 compared to AICAR, One way ANOVA with Bonferroni’s post hoc test.

**Figure 5:**
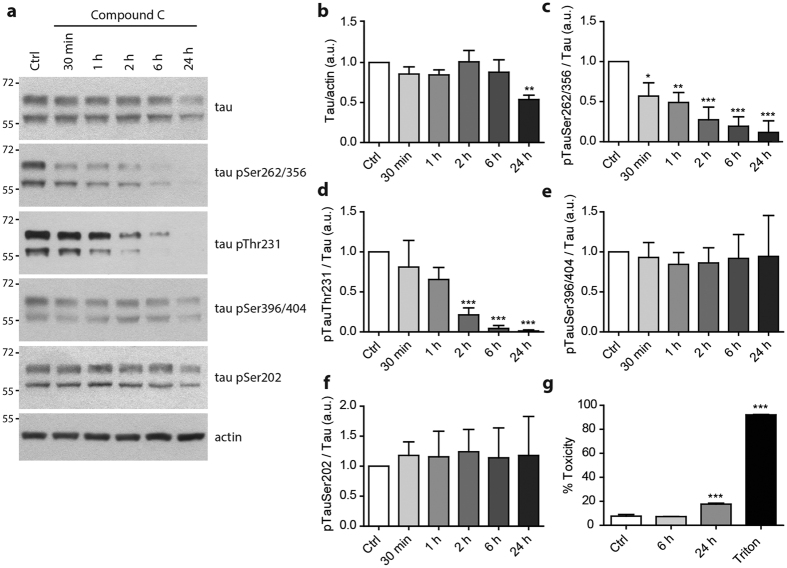
AMPK inhibition decreases tau phosphorylation in neurons. Primary neurons at 15 DIV were treated for the indicated times with the AMPK inhibitor Compound C (10 μM). WB analysis (**a**) and quantification of total tau (**b**) and phosphorylated tau at epitopes Ser^262/356^ (**c**), Thr^231^ (**d**), Ser^396/404^ (**e**) and Ser^202^ (**f**) expressed in ratios. Cytotoxicity was assessed after 6 h and 24 h of Compound C treatment using LDH assay (**g**). Results represent mean ± SD, n = 4–6. a.u., arbitrary units. *p < 0.05, **p < 0.01, ***p < 0.001 compared to Ctrl, One way ANOVA with Bonferroni’s post hoc test.

To rule out the possibility that the increased in tau phosphorylation following AICAR treatment could be indirectly mediated by AMPK, we used several inhibitors of the main known tau kinases. Primary neurons were pre-treated with AMPK, MARK1, cdk5, MEK, PKA, mTOR and PI3K inhibitors before being subjected to AICAR treatment. Phosphorylation of ACC, the direct substrate of AMPK, was used as a control of AMPK inhibition. Except for the co-treatment with Compound C, an AMPK inhibitor, neither of the inhibitors used could reverse the increased phosphorylation of tau that was observed following AICAR treatment (Fig. 4). In addition, Compound C was able to reduce tau phosphorylation below basal levels (Fig. 4a; control condition, lane 1) suggesting that AMPK controls basal tau phosphorylation levels. Indeed, we found that Compound C led to a rapid decrease in tau phosphorylation levels (Fig. 5a–f) at the same epitopes that were found to be phosphorylated following AMPK activation. These results suggest that AMPK is one of the main tau kinase phosphorylating tau at the epitopes Ser^262/356^ and Thr^231^ at basal conditions in neurons. While after 24 h of treatment we observed a slight toxicity of Compound C, this toxicity could not be solely responsible for the decrease of tau phosphorylation since Compound C was not toxic up to 6 h of treatment (Fig. 5g) and the decreased phosphorylation of tau was already observed after 30 min of treatment.

### AMPKα2 deficiency reduces endogenous tau phosphorylation

**Figure 6:**
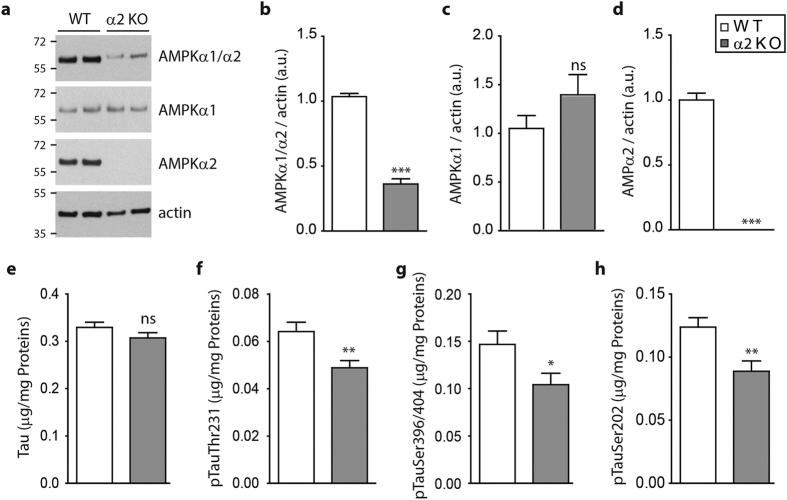
Endogenous levels of phosphorylated tau in AMPK α2 KO brains. WB analysis (**a**) and quantification of AMPKα1/α2 (**b**), AMPKα1 (**c**) and AMPKα2 (**d**) expressed in ratio of actin. Results represent mean ± SEM, n = 6 (WT) and 6 (α2 KO) of 8-months old WT and AMPKα2^−/−^ (α2 KO) mouse brains. (**e–h**) ELISA quantifications of endogenous total tau (**e**) and phosphorylated tau at epitopes Thr^231^ (**f**), Ser^396/404^ (**g**) and Ser^202^ (**h**) in heat stable fraction of 6-months old WT and AMPKα2^−/−^ (α2 KO) mouse brains. Results represent mean ± SEM, n = 17 (WT) and 20 (α2 KO); *p < 0.05, **p < 0.01, ***p < 0.001 (Student’s t test). a.u., arbitrary units.

To go further and to determine whether, *in vivo*, AMPK was also involved in tau phosphorylation, we assessed endogenous tau phosphorylation in mice that were deficient for one of the two AMPK catalytic subunits, the AMPKα2^−/−^ mice^35^. Using subunits specific antibodies, we confirmed that the AMPKα2 subunit was the predominant subunit in the mouse brain, accounting for at least 65% of total AMPKα (Fig. 6a,b,d). Additionally, while there is a slight increase in AMPKα1 subunit levels, it did not compensate for the loss of the AMPKα2 subunit (Fig. 6a,c). To quantitatively assess different epitopes of tau relevant to AD, we used ultra-sensitive ELISAs^36^. Using these previously well characterized highly sensitive assays we observed a decrease of endogenous tau phosphorylation in the brain of the AMPKα2^−/−^ compared to wild type mice while no significant differences were observed for the levels of total tau (Fig. 6e–h). These results suggest that AMPK can also regulate endogenous tau phosphorylation *in vivo*.

### AMPKα2 deficiency reduces tau pathology in PS19 transgenic mice

**Figure 7:**
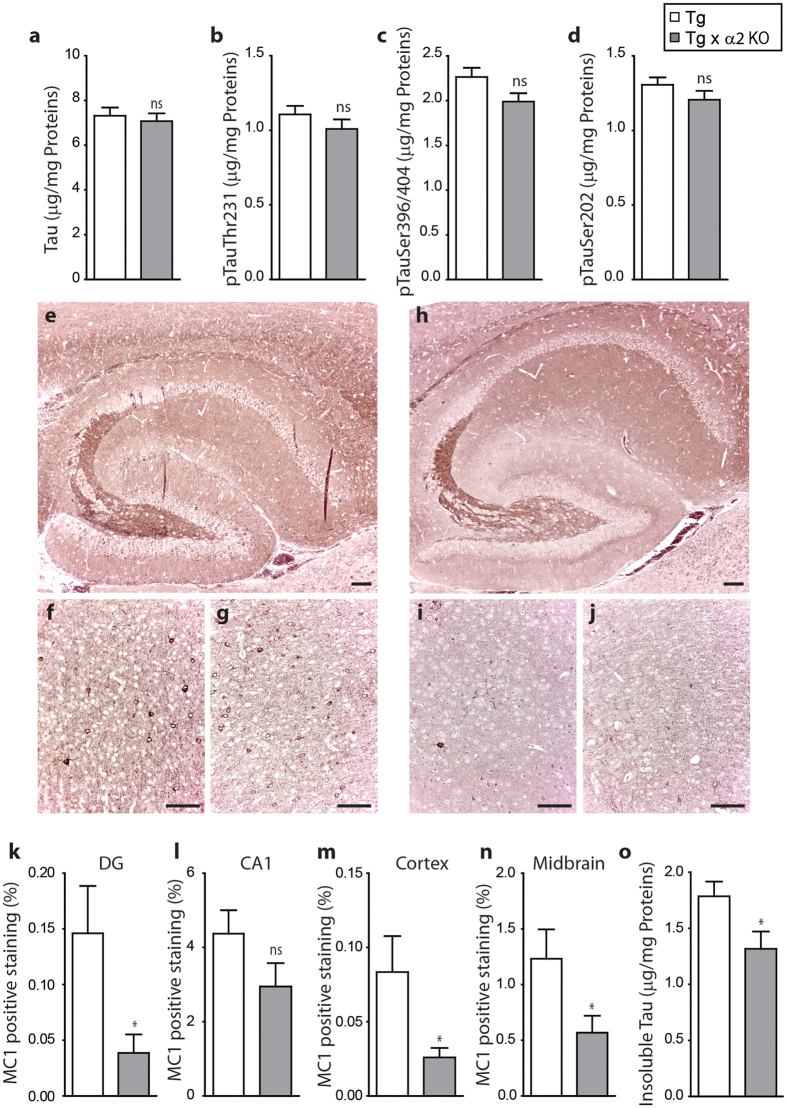
Impact of AMPK α2 deficiency on tau pathology. (**a–d**) ELISA quantifications of total tau (**a**) and phosphorylated tau at epitopes Thr^231^ (**b**), Ser^396/404^ (**c**) and Ser^202^ (**d**) in heat stable fraction of 8-months old AMPKα2^+/+^: tau^P301S^ (Tg) and AMPKα2^−/−^:tau^P301S^ (Tg × α2 KO) mouse brains. (**e–j**) Immunohistochemistry of MC1 in Tg (**e–g**) and Tg × α2 KO (**h–j**) mouse brains in hippocampus (×5) (**e,h**), cortex (×20) (**f,i**) and midbrain (× 20) (**g**,**j**). Scale bars = 100 μm. (**k–n**) Quantifications of MC1 positive staining in dentate gyrus (DG, **k**) CA1 (**l**) cortex (**m**) and midbrain (**n**) expressed in percentage. Results represent mean ± SEM, n = 6, *p < 0.05 (Student’s t test). (**o**) ELISA quantifications of total tau in insoluble fraction of Tg and Tg × α2 KO mouse brains. Results represent mean ± SEM, n = 22, *p < 0.05 (Student’s t test).

In order to determine if AMPKα2 deficiency could impact tau pathology and tangle formation *in vivo*, AMPKα2^−/−^ mice were crossed with the PS19 tau transgenic mice^37^. The resulting AMPKα2^−/−^:tau^P301S^ mice were compared to AMPKα2^+/+^:tau^P301S^ mice. Heat stable and insoluble tau fractions were obtained from 8 months old animals and analyzed by ELISA as previously described^38^. We did not observe any significant effect of AMPKα2 deficiency on total or phosphorylated tau levels in the heat-stable preparations (Fig. 7a–d and Fig. S3). However, we observed a significant decrease of the conformational tau epitope MC1 staining in the hippocampus, cortex, and midbrain of the AMPKα2^−/−^:tau^P301S^ mice as compared to AMPKα2^+/+^:tau^P301S^ mice (Fig. 7e–n). This decrease of MC1 staining was accompanied by a significant reduction in insoluble total tau in the AMPKα2^−/−^:tau^P301S^ mice as compared to AMPKα2^+/+^:tau^P301S^ controls (Fig. 7o and Fig. S3). These results suggest that AMPK reduction could moderate tau pathology.

## Discussion

We previously demonstrated that AMPK was highly deregulated in the brain of AD and other major tauopathies patients where activated AMPK co-localized with phosphorylated tau in pre-tangle and tangle bearing neurons. An abnormal staining of AMPK was also observed in neurons not yet presenting tau hyper-phosphorylation^5^. In addition, *in vitro* studies demonstrated that AMPK was a tau kinase^5^,^23^. Together these results led us to postulate that AMPK deregulation could be an upstream event in AD and other tauopathies. In the present study, we demonstrated that AMPK was a tau kinase in primary neurons, participating in tau phosphorylation at Ser^262/356^ and Thr^231^ thereby regulating tau affinity for microtubules. In addition, we used a mouse model in which the AMPKα2 subunit was deficient to assess *in vivo* the impact of AMPK on tau phosphorylation and pathology. Concordant with the results we obtained in primary neurons, we found that AMPK could regulate endogenous tau phosphorylation *in vivo* in the mouse brain. However, we did not observe a significant decrease of tau phosphorylation in the PS19 mouse model which might be related to the presence of the P301S pathological tau mutation. Indeed, tau phosphorylation status is altered by the presence of pathological mutations^39^. It is interesting to note that phosphorylation of the Ser^202^ and Ser^396/404^ epitopes that were not or slightly regulated by AMPK *in vitro* or in primary neurons were found to be significantly decreased *in vivo* in the brain of AMPKα2 deficient mice. Therefore it is possible that, *in vivo*, AMPK could also indirectly regulate tau phosphorylation. Indeed, Thr^231^ phosphorylation was suggested to induce local conformational change in tau that would alter the inhibitory activity of the N-terminus of tau. As a consequence, Thr^231^ might serve as a priming site for GSK3β^40^, thereby allowing for increased tau phosphorylation at GSK3β epitopes including Ser^396/404^. In addition, our results show that the phosphorylation pattern of tau Thr^231^ is different from that of p-Thr^172^AMPK following AICAR treatment. These results suggest that tau Thr^231^ phosphorylation induced by AMPK activation might be transient. It is possible that phosphorylation at this site is also controlled by other mechanisms, including dephosphorylation. Indeed, Thr^231^ was described to be the target for Pin1, a peptidyl-cis-trans isomerase that was reported to favor Thr^231^ dephosphorylation by PP2A^41^,^42^. It is also possible that AICAR and metformin might be regulating other pathways that would eventually lead to tau dephosphorylation. It was shown for example that metformin induces tau dephosphorylation by directly activating PP2A^43^. In addition, a recent study showed that AMPK phosphorylation at Ser^485^, which is believed to be an inhibitory phosphorylation site, was correlated with tau dephosphorylation^28^. Therefore it is possible, that AICAR and/or metformin could activate AMPK phosphorylation at Ser^485^ (for AMPKα1 subunit) or Ser^491^ (for AMPKα2 subunit) thereby inactivating the kinase and AMPK-mediated tau phosphorylation. While this phosphorylation was reported to be necessary for AMPK inhibition, it is not sufficient^44^. Overall, our results could in part explain the discrepancies that were reported in the literature, by showing that AMPK-induced tau phosphorylation is transient and might be different in regard to the cell lines that are used in which these drugs might differently affect non-AMPK dependent pathways.

While our *in vivo* studies have only assessed the impact of AMPKα2 subunit deletion on tau phosphorylation, we cannot exclude the possibility that the AMPKα1 subunit might also be involved in tau phosphorylation, and further studies using AMPKα1 KO mice are warranted. It will also be interesting to determine to which extend tau pathology is modulated in double AMPKα1/α2 KO mice. Unfortunately the double AMPKα1/α2 KO is embryonic lethal^45^ and neuron-specific conditional KO models will be required.

In conclusion, our present study suggests that AMPK mediates tau phosphorylation in neurons and *in vivo* in the mouse brain. AMPK shares at least the same properties as the main tau kinases that act in AD. Indeed, evidence suggests that AMPK participates in APP processing^32^,^46^,^47^, synaptic dysfunction^24^,^25^,^26^, neuronal death^48^,^49^ and tau phosphorylation^5^,^23^ (this study). Finally, given that glucose hypometabolism and mitochondrial defects are described to occur early in the course of the disease, it is likely that AMPK, which is directly regulated by energy status, could be an early player during the development of the pathology.

## Methods

### Antibodies and reagents

Antibodies directed against AMPKα1/α2, ACC, pSer^79^-ACC were obtained from Cell Signaling Technology. Anti-AMPK α1 and anti-AMPK α2 antibodies were from R&D Systems. Anti p-Thr^172^AMPK antibody and p-Thr^172^AMPK blocking peptide were obtained from Santa Cruz Biotechnology^24^. Anti-actin antibody was from BD Transduction Laboratory. Anti-MAP2, anti-α-tubulin and anti-acetylated-α-tubulin antibodies were from Sigma. Mouse monoclonal antibodies PHF1 (tau pSer^396^/Ser^404^)^50^, CP13 (tau pSer^202^)^51^, 2E12 or RZ3 (tau pThr^231^)^5^, DA9 (total tau aa102-140)^36^, DA31 (total tau aa220-240)^36^ and MC1 (conformation dependent antibody that recognizes only tau in a pathological conformation)^52^ were previously described. 12E8 monoclonal antibody was obtained from Dr. Seubert (Elan Pharmaceuticals, San Francisco, CA; tau pSer^262/356^)^53^. As total tau antibody in the Western-blot experiments, we used tau Cter (homemade well characterized antibody recognizing the 11 amino-acids in the c-terminal part of tau27)^54^. Tau5 (total tau) and AT180 (tau pThr231) were from Invitrogen.

AICAR, metformin, PD98059, H-89, LY294002 were purchased from Tocris; roscovitin and MARK/Par1 inhibitor from Merck; Compound C was from Santa-Cruz Biotechnology and rapamycin was from Cell Signaling Technology.

### Animals

All animal experiments were performed according to procedures approved by the Feinstein Institute for Medical Research Institutional Animal Care and Use Committee. PS19 tau transgenic mice expressing P301S human mutant tau protein under the direction of the mouse prion protein promoter were obtained from The Jackson Laboratory (Bar Harbor, ME, USA)^37^ and were crossed with the AMPKα2 knock-out mice (AMPKα2^−/−^)^35^ to obtain AMPKα2^−/−^:Tau^P301S^ and AMPKα2^+/+^:Tau^P301S^ controls. Males and females were used at 6 or 8 months of age as indicated. Following sacrifice, brains were quickly isolated, and hemi-brains were either snap-frozen or immersion-fixed in 4% paraformaldehyde (PFA) for histochemical analyses.

### Tissue preparation

Brains were homogenized in an appropriate volume of homogenizing buffer, a solution of TBS (Tris Buffered Saline), pH 7.4 containing 10 mM NaF, 1 mM NaVO_3_, 2 mM EGTA, proteases and phosphatases inhibitors (complete Mini Roche). Protein concentrations were determined using BCA assay.

For heat stable tau, NaCl was added to a 500 μl aliquot of homogenate to a final concentration of 250 mM, and β-Mercaptoethanol was added to 5% final concentration. Homogenates were mixed, heated at 100 °C for 15 minutes, mixed again by vortexing, cooled on ice for 30 minutes, vortexed again and spun down at 20,000 × g for 10 minutes at 4 °C. The supernatant, containing heat stable tau fraction was retained and used for subsequent experiments.

To obtain insoluble tau preparation^38^, homogenates were spun at 6,000 × g for 10 minutes at 4 °C, supernatants were collected and spun at 200,000 × g for 30 minutes at 25 °C. Pellets were resuspended in homogenizing buffer and spun again at 200,000 × g for 30 minutes at 25 °C. Finally, pellets were resuspended in an equal amount of Laemmli sample buffer, and boiled for 5 minutes.

### Primary neuronal cultures and treatments

Primary neurons were obtained from C57BL6 wild-type mice as described previously^32^. Fetuses were obtained from females that were sacrificed at 18.5 days of gestation. Forebrains were dissected in ice-cold Hank’s balanced salt solution containing 0.5% w/v D-glucose and 25 mM Hepes under a dissection microscope. Dissociation was carried out mechanically in dissection medium containing 0.01% w/v papain, 0.1% w/v dispase II, and 0.01% w/v DNase and by incubation at 37 °C twice for 10 min. Cells were then spun down at 220 × g for 5 min at 4 °C, resuspended in Neurobasal medium supplemented with 2% B27, 1 mM sodium pyruvate, 100 units/ml penicillin, 100 μg/ml streptomycin, 2 mM Glutamax (Invitrogen), filtered through a 40-μm cell strainer, counted, and plated on poly-L-ornithine- and laminin-coated plates at a density of about 10^6^ cells/well. Culture medium was completely replaced after 16 h, and new medium (1:3 of starting volume) was added every 3 days until the end of the culture period. Drug treatments were realized directly in the conditioned media.

### Lactate dehydrogenase (LDH) release measurements

Cell toxicity was assessed by the measurement of lactate dehydrogenase release as per manufacturer’s instructions (CytoTox 96® Non-Radioactive Cytotoxicity Assay, Promega). Absorbance measurements were obtained using a SpectraMax^®^ i3 (Molecular Devices, Sunnyvale, CA 94089, USA).

### Cell fractionation into cytosolic and microtubule fractions

Cell fractionation was performed as previously described^34^. Briefly, primary neurons were recovered in warmed lysis buffer containing 1 mM MgCl_2_, 2 mM EGTA, 30% glycerol, 0.1% Triton X-100, 80 mM Pipes, pH 6.8 supplemented with proteases and phosphatases inhibitors. Homogenates were ultracentrifuged at 100,000 × g at 27 °C for 20 min, supernatants were collected as cytosolic fractions. The remaining pellets (microtubule fractions) were washed once and recovered in an equal volume to that of cytosolic fraction by sonication in RIPA buffer and homogenization at 4 °C for 1 h. Samples were mixed with loading buffer, and equal volumes were loaded and analyzed by Western blotting.

### Western blot (WB) analysis

Cell extracts were separated by SDS-PAGE and transferred to nitrocellulose membranes as described previously^32^. Membranes were probed with the antibodies listed above and analyzed by enhanced chemiluminescence detection.

### Tau enzyme-linked immunosorbent assays (ELISA)

Low tau sandwich ELISA were performed as described previously^36^. Briefly, ninety-six-well plates were coated with the following purified monoclonal tau antibodies: DA31 (total tau), PHF1 (pSer^396/404^), CP13 (pSer^202^) and 2E12 (pThr^231^) at a concentration of 6 μg/ml in coating buffer (230 mM K_2_HPO_4_, 135 mM KH_2_PO_4_, 130 mM NaCl, 2 mM EDTA, 0.05% NaN_3_, pH 7.2) for at least 48 h at 4 °C. Plates were washed 3 times in wash buffer, and blocked for 1 h at room temperature using SuperBlock (ThermoScientific). Plates were washed 5 times and 50 μl of appropriate sample, recombinant tau or PHF-tau diluted in 20% SuperBlock in 1× TBS were added to the plate. 50 μl of the total detection antibody DA9-HRP diluted 1:50 in 20% Superblock in 1× TBS was added to the samples. Plates were then incubated overnight at 4 °C. Plates were washed 9 times and 100 μl of 1-step ULTRA TMB-ELISA (ThermoScientific) was added for 30 minutes at room temperature. Finally, 100 μl of 2 M H_2_SO_4_ was added to stop the reaction, and plates were read using an Infinite m200 plate reader (Tecan) at 450 nm.

### Immunofluorescence

Neurons grown on poly-D-lysin and laminin-coated glass coverslips were fixed in 4% (PFA) and permeabilized using 0.25% Triton X-100 before blocking in 1% BSA for 1 h at RT. Neurons were then incubated with primary antibodies directed against total tau (Tau5, 1:200 dilution), MAP2 (1:200 dilution), total AMPKα1/α2 (1:100 dilution), and activated AMPK (p-Thr^172^AMPK, 1:100 dilution) O/N at 4 °C, followed with anti-IgG secondary antibodies coupled to Alexa Fluor 488 and 568 (1:500 dilution, Invitrogen). Nuclei were visualized with DAPI. Images were acquired on a Zeiss Axio Imager Z2 microscope (Carl Zeiss,Germany) equipped with a Hamamatsu ORCA-Flash4.0 digital camera (Hamamatsu Photonics, Japan) and ApoTome.2 system (Carl Zeiss,Germany). P-Thr^172^AMPK blocking peptide was used as a specificity control for the p-Thr^172^AMPK antibody. In these conditions, p-Thr^172^AMPK antibodies (200 μg/ml) were pre-incubated O/N at 4 °C with a five-fold excess of blocking peptide (1 mg/ml) in PBS (Phosphate Buffered Saline) before proceeding with the immunofluorescence protocol described above.

### *In situ* Proximity ligation assay (PLA)

To detect AMPK-tau interactions in PFA-fixed primary neurons, mouse monoclonal antibodies directed against tau (Tau5, 1:200 dilution) in combination with polyclonal rabbit antibodies directed against p-Thr^172^AMPK (1:100 dilution) were used for *in situ* proximity ligation assay. PLA was performed according to the manufacturer’s protocol (Duolink^®^ Technology). Briefly, primary antibodies were incubated as described for immunofluorescence protocol, followed by incubation with secondary antibodies conjugated to oligonucleotides strands denoted PLUS and MINUS (PLA probes Anti-Rabbit PLUS and Anti-Mouse MINUS, Sigma). The ligation and polymerization steps were conducted at 37 °C in a humid chamber followed by detection using complementary fluorescent-labeled oligonucleotides (Duolink® *In situ* Detection reagents Green). Negative controls for PLA were realized using the same conditions with either one or both of the primary antibodies lacking.

### Immunohistochemistry

Immunohistochemistry was performed as previously described^5^. Briefly, paraffin-embedded brain sections were deparaffinized by immersion in toluene and hydration through graded ethanol solutions. Antigen recovery was performed by warming the slides for 30 min at 70 °C in 10 mM citrate buffer, pH 6.0. Endogenous peroxidase activity was inhibited by incubation in 3% hydrogen peroxide for 15 min at RT. After washing in TBS containing 0.05% Triton-X100 (TBS-T), sections were blocked in 5% normal goat serum, 1 mg/ml BSA, 1 mM NaF, 0.05% Triton-X100 in TBS for 1 h at RT. Sections were then incubated in the presence of MC1 primary antibodies diluted in TBS containing 1% goat serum, 1 mg/ml BSA, 1 mM NaF and 0.05% Triton-X100 overnight at 4 °C in a humidified chamber. After washing, the sections were incubated with biotin-coupled anti-mouse secondary antibodies (1:1,000 dilution) before incubation with streptavidin-horseradish peroxidase (1:1,000 dilution, Southern Biotech) and visualization with diaminobenzidine tetrahydrochloride. Staining was performed blindly on 3 slices per animal. Images were quantified using a constant threshold on Mercator software (Explora Nova) to obtain the ratio of the surface stained on the surface of the region of interest.

### Statistical analysis

Group comparisons were made using one-way ANOVA with Bonferroni’s *post-hoc* test using GraphPad Prism (Prism 5.0d, GraphPad Software Inc, La Jolla, CA, USA). All data are reported as mean ± SD or SEM, and a value of p < 0.05 was considered statistically significant.

## Additional Information

**How to cite this article**: Domise, M. *et al.* AMP-activated protein kinase modulates tau phosphorylation and tau pathology *in vivo. Sci. Rep.* **6**, 26758; doi: 10.1038/srep26758 (2016).

## Supplementary Material

Supplementary Information
