# Supplementary material for: AMP-activated protein kinase modulates tau phosphorylation and tau pathology in vivo
Source: Sci Rep. 2016 May 27;6:26758. doi: 10.1038/srep26758 (PMC4882625; doi:10.1038/srep26758)
Supplement: Supplementary Information [file srep26758-s1.pdf]

Title:

AMP-activated protein kinase modulates tau phosphorylation and tau pathology *in vivo*

Authors:

Manon Domise[1], Sébastien Didier[1], Claudia Marinangeli[1], Haitian Zhao[2], Pallavi Chandakkar[2], Luc Buée[1], Benoit Viollet[3,4,5], Peter Davies[2], Philippe Marambaud[2], Valérie Vingtdeux[1*]

Figure S1. p-Thr^{172}AMPK staining specificity

a. Primary neurons at 15 DIV were treated with the AMPK activator AICAR (1mM, 6 h) and labeled with p-Thr^{172}AMPK (pAMPK) antibody using identical acquisition settings. **b.** Immunofluorescence staining of p-Thr^{172}AMPK without (top) or with (bottom) p-Thr^{172}AMPK blocking peptide in primary neurons at DIV 15 using identical acquisition settings. Scale bars = 50 µm.

Figure S2. Metformin-mediated AMPK activation induces tau phosphorylation in primary neurons

Primary neurons at 15 DIV were treated for the indicated times with metformin (2.5 mM). AMPK and ACC expression and activation were monitored by WB using antibodies against AMPK, p-Thr^{172}AMPK (pAMPK), ACC, p-Ser^{79}ACC (pACC) and actin (**a**). Quantifications of the ratios AMPK/actin (**b**) pAMPK/AMPK (**c**) and pACC/ACC (**d**). WB analysis (**e**) and quantification of total tau (**f**) and phosphorylated tau at epitopes Ser$^{262/356}$ (**g**), Thr^{231} (**h**), Ser$^{396/404}$ (**i**) and Ser^{202} (**j**) expressed in ratios. LDH cytotoxicity assay following 6 h and 24 h metformin treatments (**k**). Results represent mean ± SD, n=4-6. a.u., arbitrary units. * $p<0.05$, ** $p<0.01$, *** $p<0.001$ compared to Ctrl, One way ANOVA with Bonferroni's post hoc test.

Figure S3. Western-blot analysis of tau in heat-stable, soluble and insoluble fractions

WB analysis of total tau and phosphorylated tau at epitopes Thr^{231}, Ser$^{396/404}$ and Ser^{202} in heat stable (**a**), soluble (**b**), and insoluble (**c**) fractions of 8-months old AMPK$\alpha 2^{+/+}$:tau^{P301S} (Tg) and AMPK$\alpha 2^{-/-}$:tau^{P301S} (Tg x α2 KO) mouse brains.

Figure S1

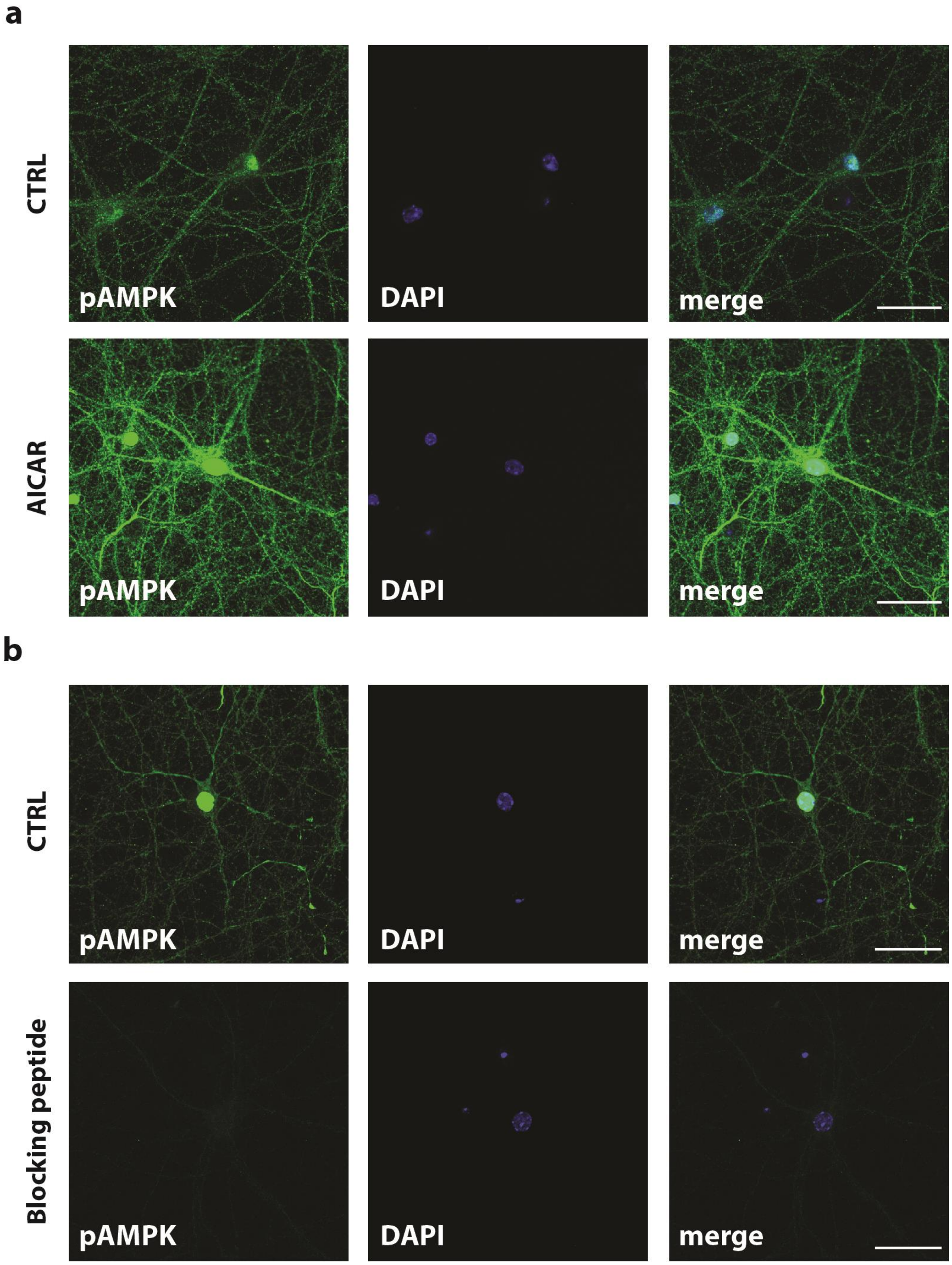
a
CTRL
AICAR
pAMPK
DAPI
merge
b
CTRL
Blocking peptide
pAMPK
DAPI
merge

Figure S2

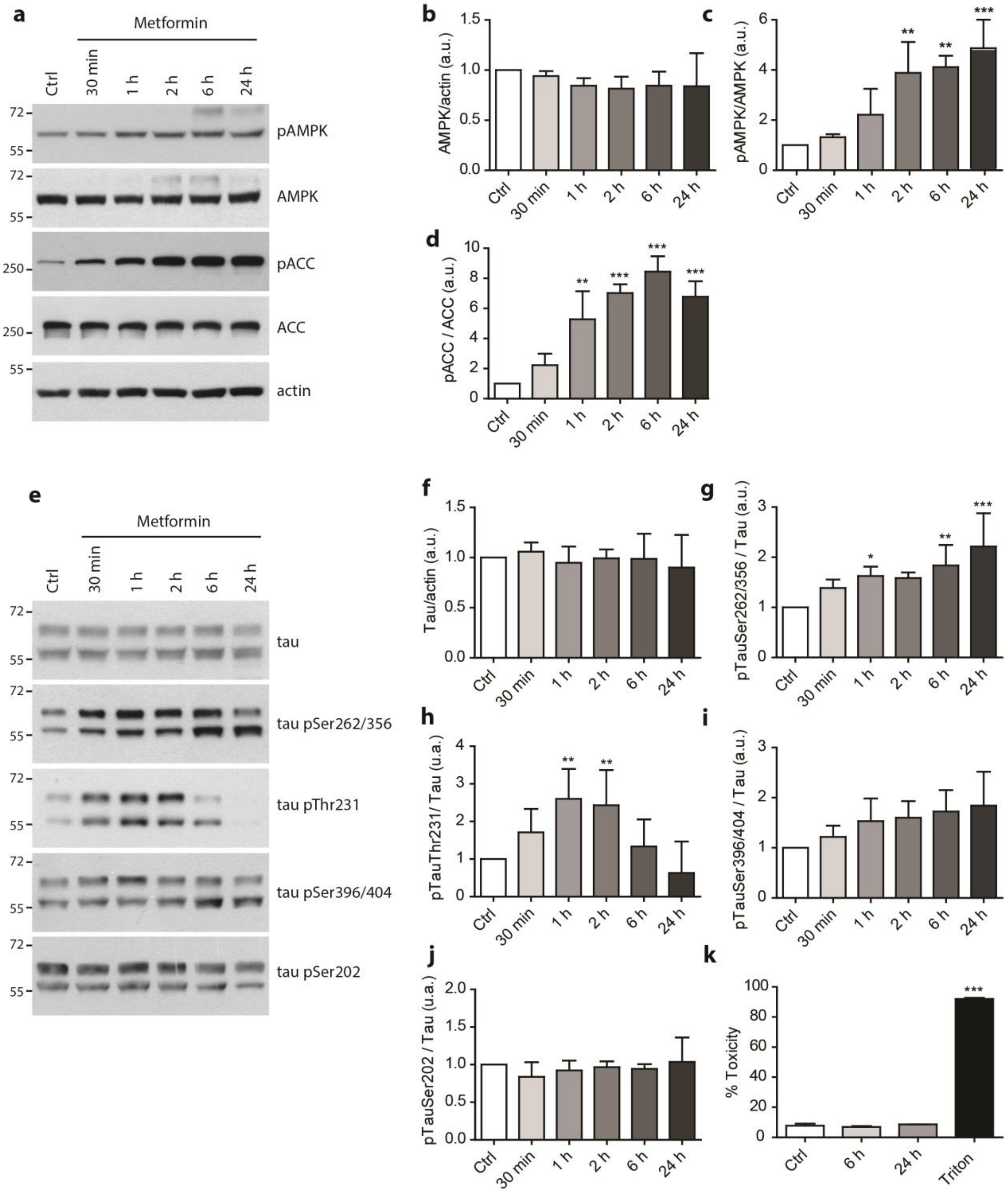
a
Metformin
Ctrl
30 min
1 h
2 h
6 h
24 h
pAMPK
AMPK
pACC
ACC
actin
b
AMPK/actin (a.u.)
c
pAMPK/AMPK (a.u.)
d
pACC / ACC (a.u.)
e
tau
tau pSer262/356
tau pThr231
tau pSer396/404
tau pSer202
f
Tau/actin (a.u.)
g
pTauSer262/356 / Tau (a.u.)
h
pTauThr231/ Tau (u.a.)
i
pTauSer396/404 / Tau (a.u.)
j
pTauSer202 / Tau (u.a.)
k
% Toxicity
Triton

Figure S3

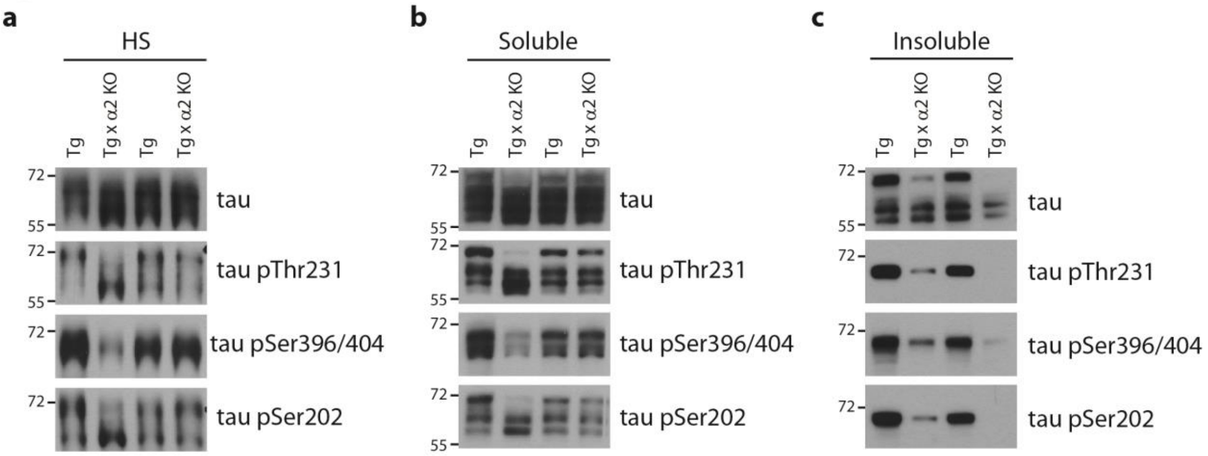
a
HS
Tg
Tg x α2 KO
Tg
Tg x α2 KO
72
55
tau
72
55
tau pThr231
72
tau pSer396/404
72
tau pSer202
b
Soluble
Tg
Tg x α2 KO
Tg
Tg x α2 KO
72
55
tau
72
55
tau pThr231
72
tau pSer396/404
72
55
tau pSer202
c
Insoluble
Tg
Tg x α2 KO
Tg
Tg x α2 KO
72
55
tau
72
tau pThr231
72
tau pSer396/404
72
tau pSer202
